# Supplementary material for: An Arabidopsis AT-hook motif nuclear protein mediates somatic embryogenesis and coinciding genome duplication
Source: Nat Commun. 2021 May 4;12:2508. doi: 10.1038/s41467-021-22815-8 (PMC8096963; doi:10.1038/s41467-021-22815-8)
Supplement: Supplementary file 1 — Supplementary Information [file 41467_2021_22815_MOESM1_ESM.pdf]

Supplementary Information

An *Arabidopsis* AT-hook motif nuclear protein mediates somatic embryogenesis and coinciding genome duplication

Omid Karami[1]*, Arezoo Rahimi[1], Patrick Mak[1,4], Anneke Horstman[2,3], Kim Boutilier[2], Monique Compier[1,5], Bert van der Zaal[1] and Remko Offringa[1]*

[1]Plant Developmental Genetics, Institute of Biology Leiden, Leiden University, Sylviusweg 72, 2333 BE Leiden, Netherlands

[2]Bioscience, Wageningen University and Research, Droevendaalsesteeg 1, 6708 PB Wageningen, Netherlands

[3]Molecular Biology, Wageningen University and Research, Droevendaalsesteeg 1, 6708 PB Wageningen, Netherlands

[4]Current address: Sanquin Plasma Products B.V., Plesmanlaan 125, 1066 CX Amsterdam, Netherlands

[5]Current address: Rijk Zwaan Netherlands B.V., Burgemeester Crezéelaan 40, P.O. Box 40, 2678 ZG De Lier, The Netherlands

*Correspondence should be addressed to O.K. (o.karami@biology.leidenuniv.nl) and R.O. (r.offringa@biology.leidenuniv.nl)

Supplementary Figures 1 to 19

Supplementary Table 1

Running title: Somatic embryogenesis and genome duplication

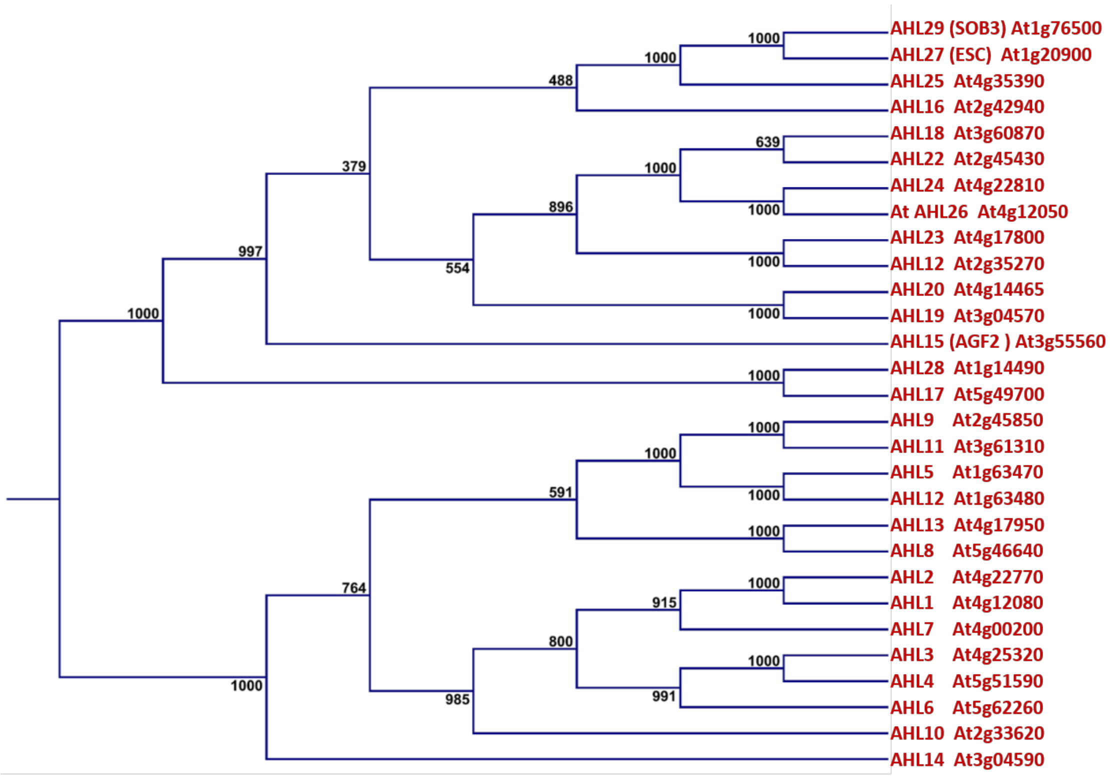

Supplementary Figure 1 A phylogenetic tree of the *Arabidopsis AHL* gene family.

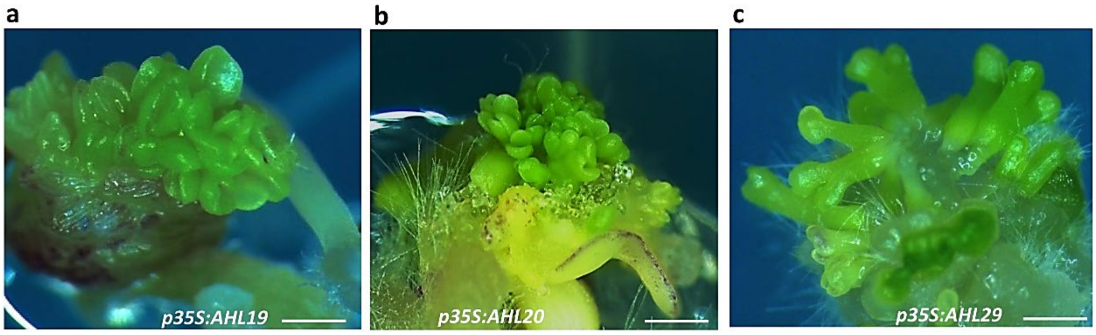

Supplementary Figure 2 Overexpression of *AHL19, AHL20 and AHL20* induces SE. a-c The embryo structures induced on IZEs of *p35S:AHL19* (**a**), *p35S:AHL20* (**b**) or *p35S:AHL29* (**c**) plants cultured for 2 weeks on medium lacking 2,4-D. Size bar indicates 1 mm.

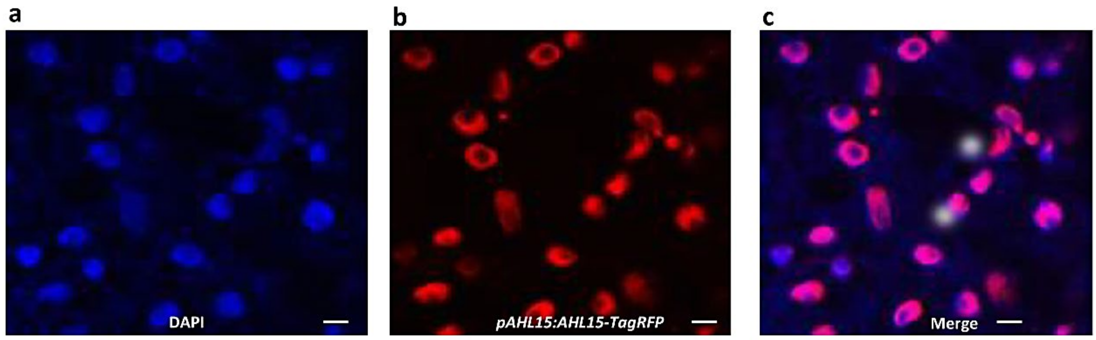

Supplementary Figure 3 Nuclear localization of AHL15 in embryo cells. a-c Confocal images of embryo cells in torpedo stage. The blue channel showing nuclear staining by DAPI (**a**), the RFP channel showing nuclear-localized AHL15-tagRFP (**b**), and the merged images (**c**). Size bar indicates 4 µm. Similar results were obtained from 4 independent experiments.

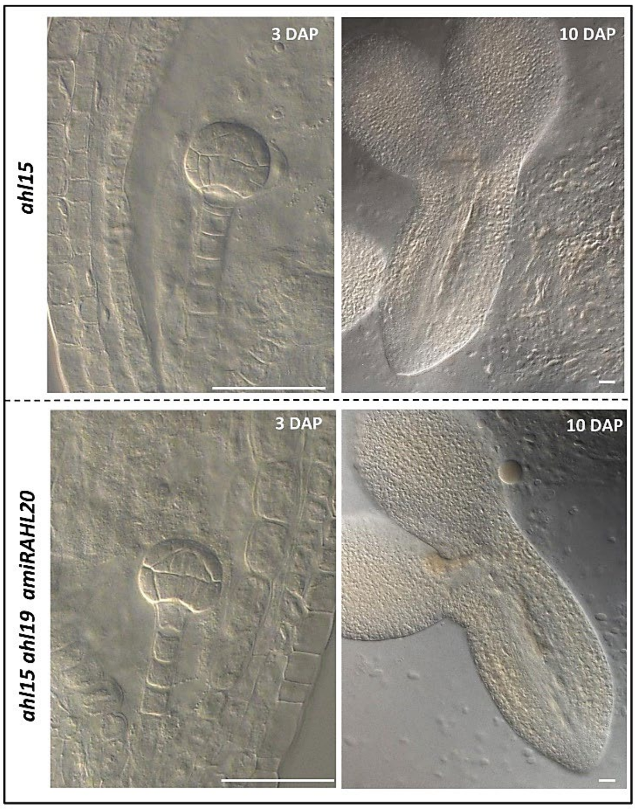

Supplementary Figure 4 ***AHL*** **genes act redundantly during zygotic embryogenesis.** DIC images of zygotic embryo development in siliques of *ahl15* (upper panel) or *ahl15 ahl19 amiRAHL20* (lower panel) plants at 3 or 10 days after pollination (DAP). Size bar indicates 40 µm. Similar results were obtained from 3 independent experiments.

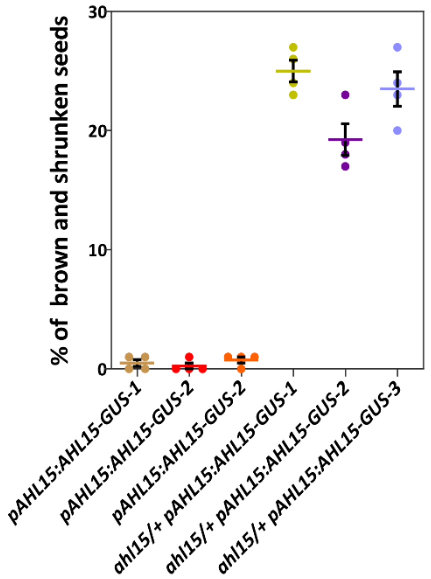

Supplementary Figure 5 Aberrant seed development in *ahl15/+* plants expressing the dominant negative *pAHL15:AHL15-GUS* fusion. Percentage of brown, shrunken seeds in *pAHL15:AHL15-GUS* and *ahl15/+ pAHL15:AHL15-GUS* siliques. Dots indicate the values of 4 biological replicates per plant line with about 200 seeds in 3-4 siliques scored per replicate, bar indicates the mean, and error bars the s.e.m.

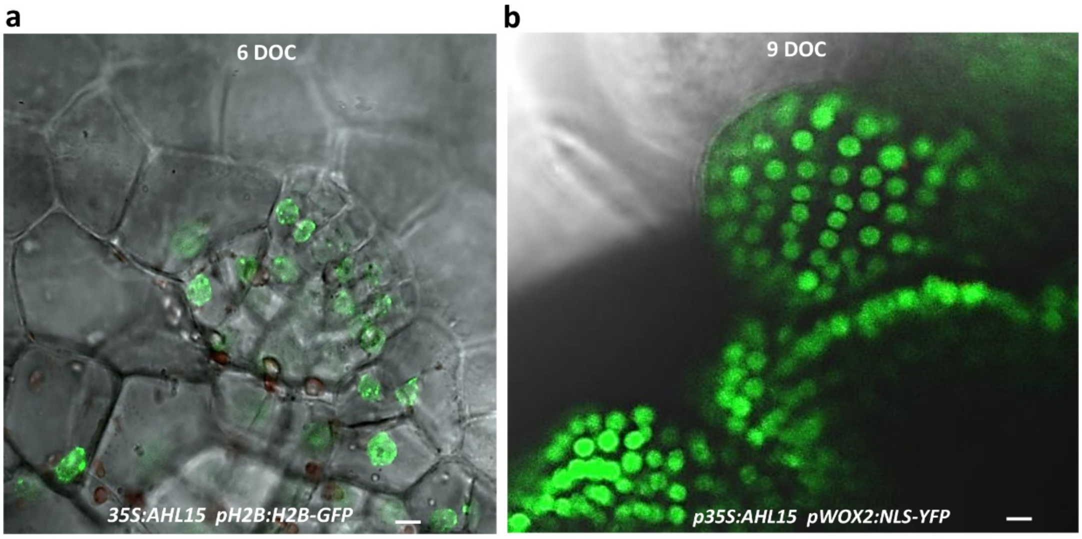

Supplementary Figure 6 Early cell division events and somatic embryos on cotyledons of *p35S:AHL15* IZEs. a Visualization of cell division using H2B-GFP labelling in adaxial protodermal cotyledon cells of an *p35S:AHL15* IZE after 6 days of culture (DOC). Similar images were obtained from 3 independent experiments. **b** Visualization of pro-embryos using the *pWOX2:NLS-YFP* embryo marker on the adaxial side of cotyledons of an *p35S:AHL15* IZE after 9 DOC. Size bar indicates 7 µm. Similar images were obtained from 5 independent experiments.

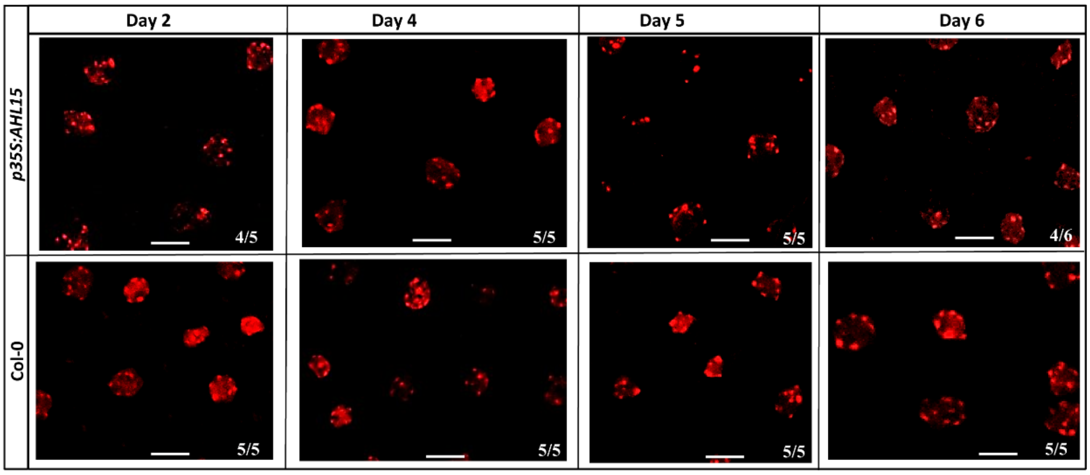

Supplementary Figure 7 No clear heterochromatin decondensation in wild-type or *35S::AHL15* IZEs in the first 6 days of culture. Visualization of heterochromatin condensation using propidium iodide staining of nuclei in cotyledon protodermal cells of wild-type or *p35S:AHL15* IZEs 2, 4, 5 or 6 DOC. Size bar indicates 6 µm, the numbers indicate the frequency with which similar images were obtained from 3 independent experiments. Deviating images showed reduced heterochromatin condensation.

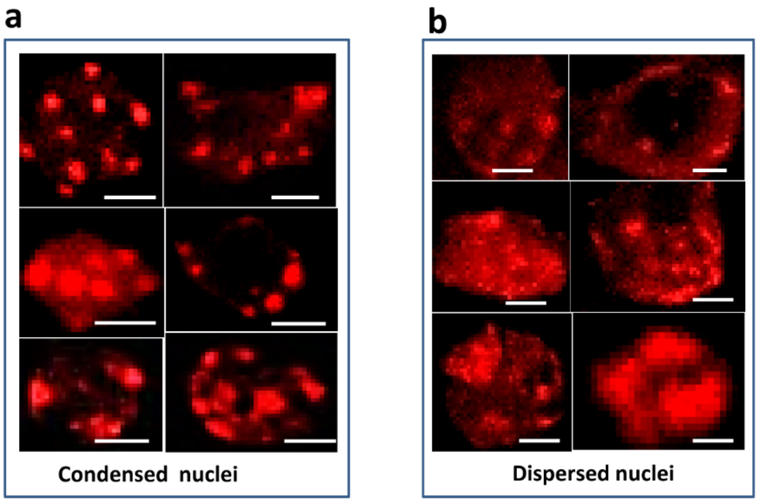

Supplementary Figure 8 Classification of the chromatin phenotypes of propidium iodide-labelled nuclei. a, b Propidium iodide stained nuclei of protodermal cells at the adaxial side of cotyledons of wild-type and *p35S:AHL15* IZEs, categorized into condensed nuclei (**a**) and dispersed nuclei (**b**). Size bar indicates 2.5 µm.

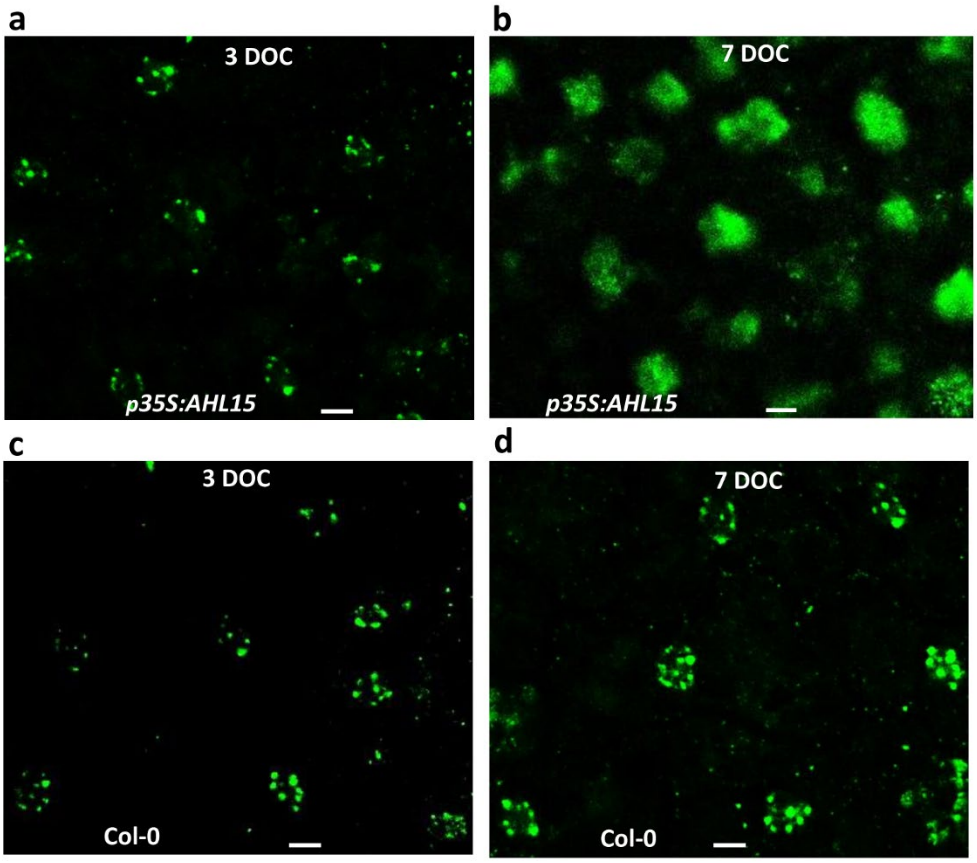

Supplementary Figure 9 Visualization of heterochromatin compaction in nuclei of cotyledon protodermal cells. **a-d** Confocal microscopy images of protodemal cells at the adaxial side of cotyledons of wild-type (**c**, **d**, Col-o) and *p35S:AHL15* (**a**, **b**) IZEs that were immunostained for the heterochromatin marker H3K9me2after 3 (**a**, **c**) or 7 (**b**, **d**) days of culture (DOC). Size bar indicates 5 µm. Similar results were obtained from 2 independent experiments.

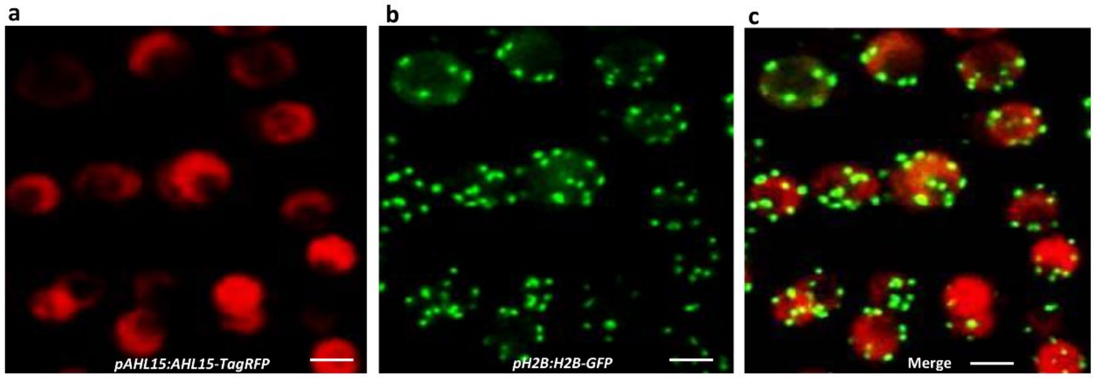

Supplementary Figure 10 AHL15-tagRFP does not colocalize with H2B-GFP-marked heterochromatin. a-c Confocal images of root meristem cells. The RFP channel showing nuclear-localised AHL15-tagRFP (**a**), the GFP channel showing H2B-GFP marked heterochromatin (**b**), and the merged image (**c**). Size bar indicates 5 µm. Similar results were obtained from 3 independent experiments.

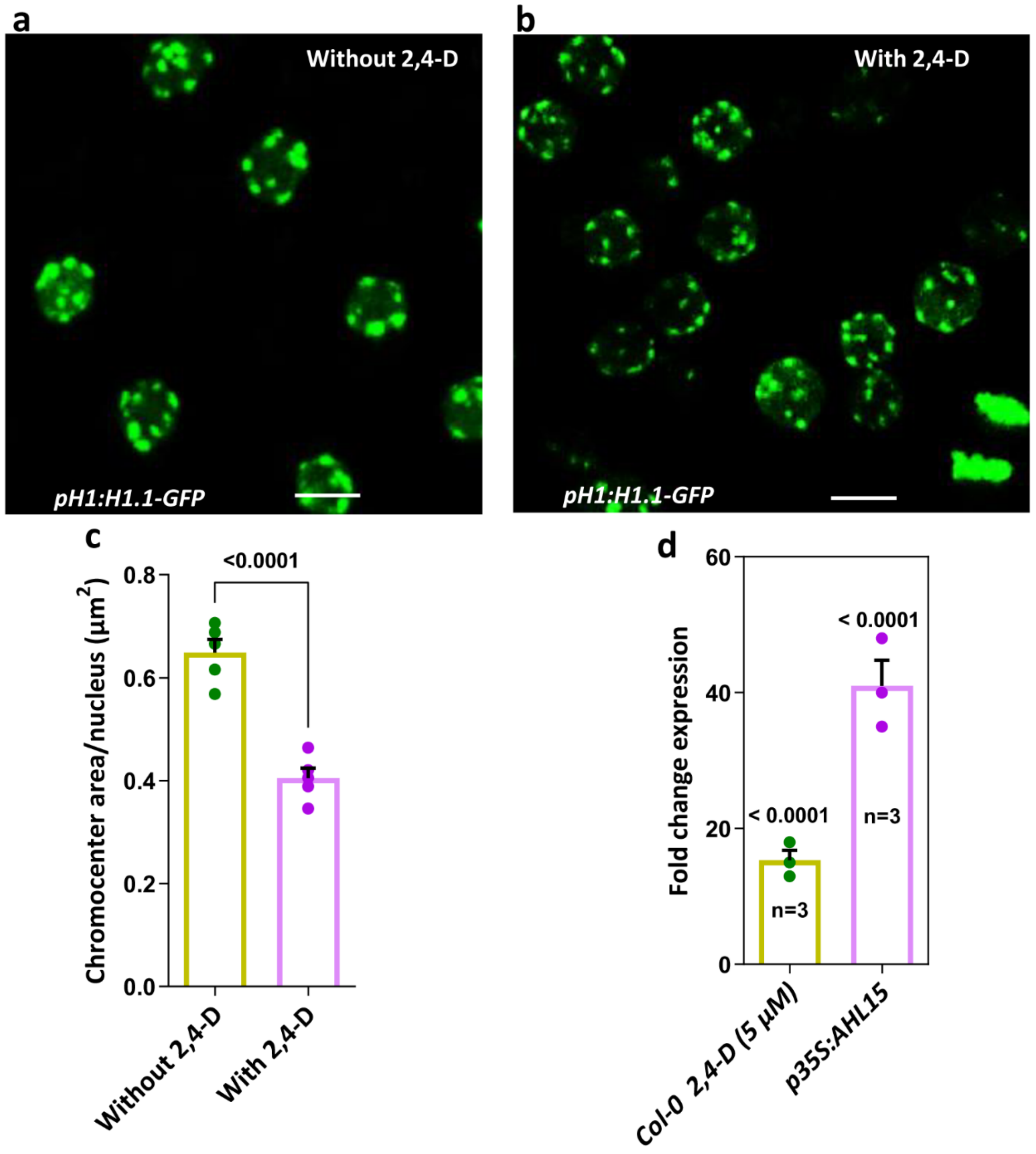

Supplementary Figure 11 2,4-D treatment only results in slight heterochromatin decondensation. a, b Heterochromatin condensation, as visualized by H1.1-GFP labelled nuclei in protodermal cells at the adaxial side of cotyledons of *H1.1:H1.1-GFP* IZEs, 7 days after culture on B5 medium containing 2,4-D (**a**) or on B5 medium without 2,4-D (**b**). Size bar indicates 5 µm. **c** Quantification of the H1.1-GFP-labelled chromocenter area in nuclei of cotyledon cells as shown in **a** and **b**. Dots indicate the values of six biological replicates per treatment with measurement of 30 chromocenters pre replicate (2 or 3 of the most clear chromocenters per nucleus), bar indicates the mean, and error bars the s.e.m. The p-value was determined by a two-sided Student's *t*-test. **d** *AHL15* expression in wild-type 2,4-D treated IZEs and in *p35S:AHL15* IZEs for 7 days relative to that in wild-type IZEs on B5 medium without 2,4-D. Error bars indicate the standard error of the mean of three biological replicates. Dots indicate the values of three biological replicates, bar indicates the mean, and error bars the s.e.m. The p-values were determined by a two-sided Student's *t*-test.

.

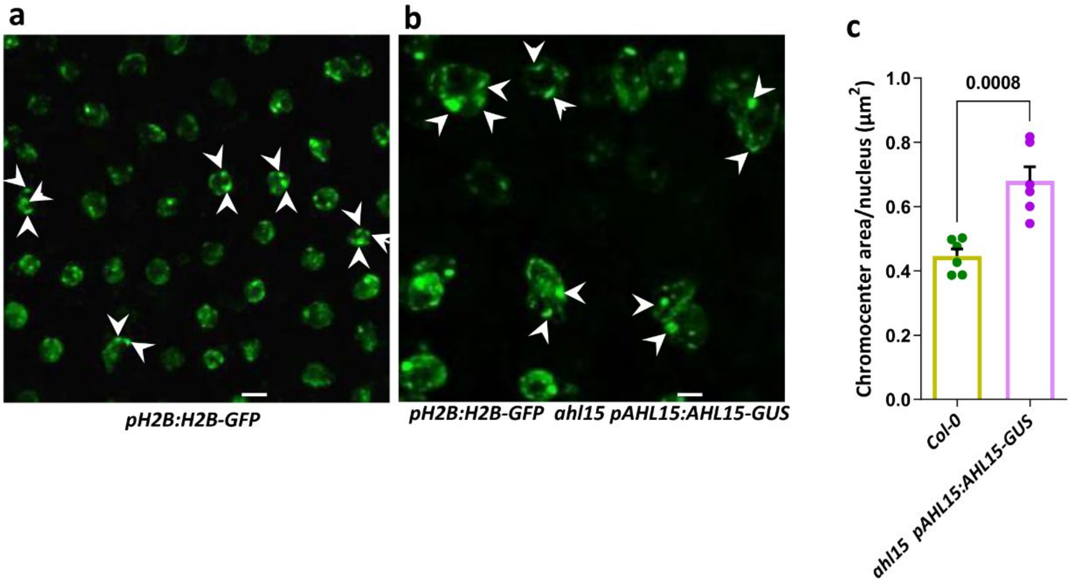

Supplementary Figure 12 AHL proteins reduce chromocenter size during ZE. **a, b** Visualization of chromocenters using the *pH2B:H2B-GFP* reporter in wild-type (**a**) and defective *ahl15 pAHL15:AHL15-GUS* ZEs (**b**) at 6 DAP. Size bar indicates 3.5 µm. **c** Quantification of the chromocenter area labelled with H2B-GFP in nuclei of wild-type and *ahl15 pAHL15:AHL15-GUS* ZEs at 6 DAP. Dots indicate the values of six biological replicates per plant line with measurement of 30 chromocenters pre replicate (2 or 3 of the most clear chromocenters per nucleus), bar indicates the mean, and error bars the s.e.m. The p-value was determined by a two-sided Student's *t*-test.

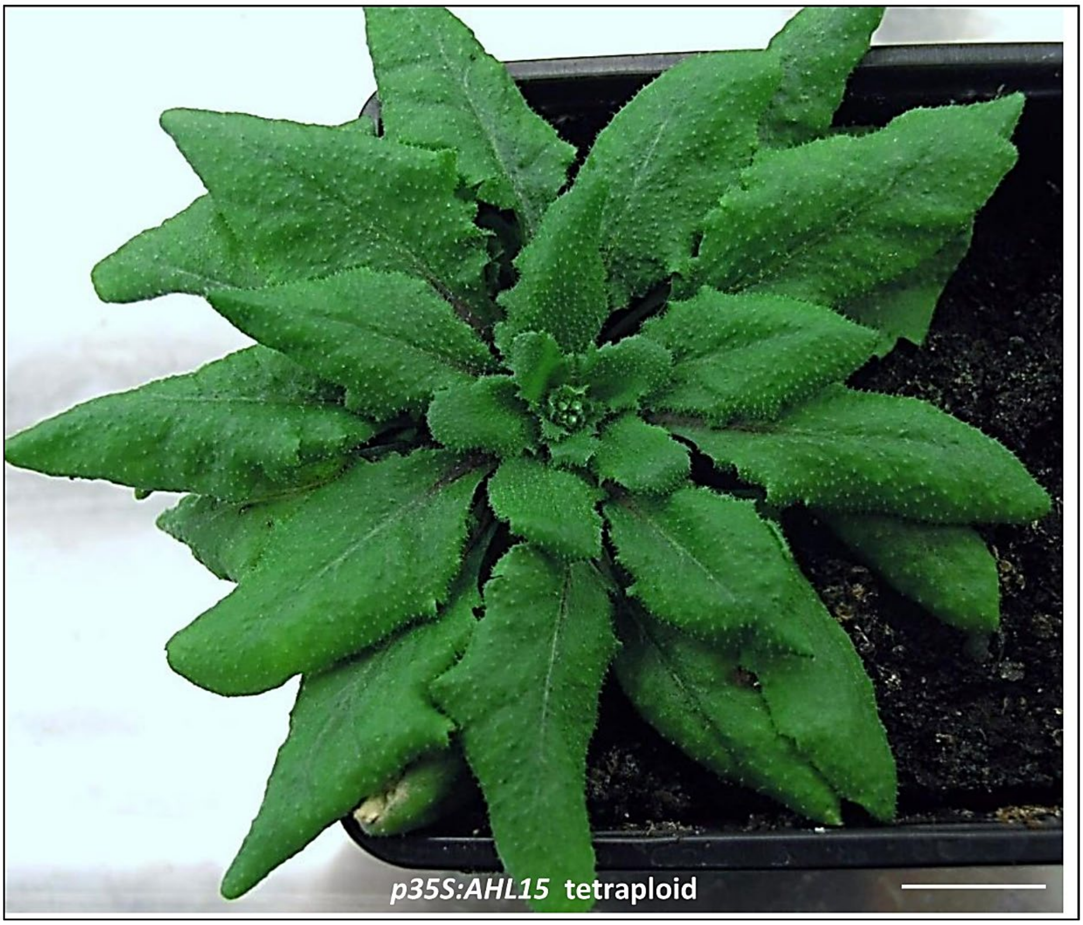

Supplementary Figure 13 Phenotype of a SE-derived polyploid *p35S:AHL15* plant. Dark green leaves and large rosette of tetraploid plants regenerated from *p35S:AHL15*-induced somatic embryos, grown in long day conditions (16 hr light/ 8 hr dark). Size bar indicates 1 cm.

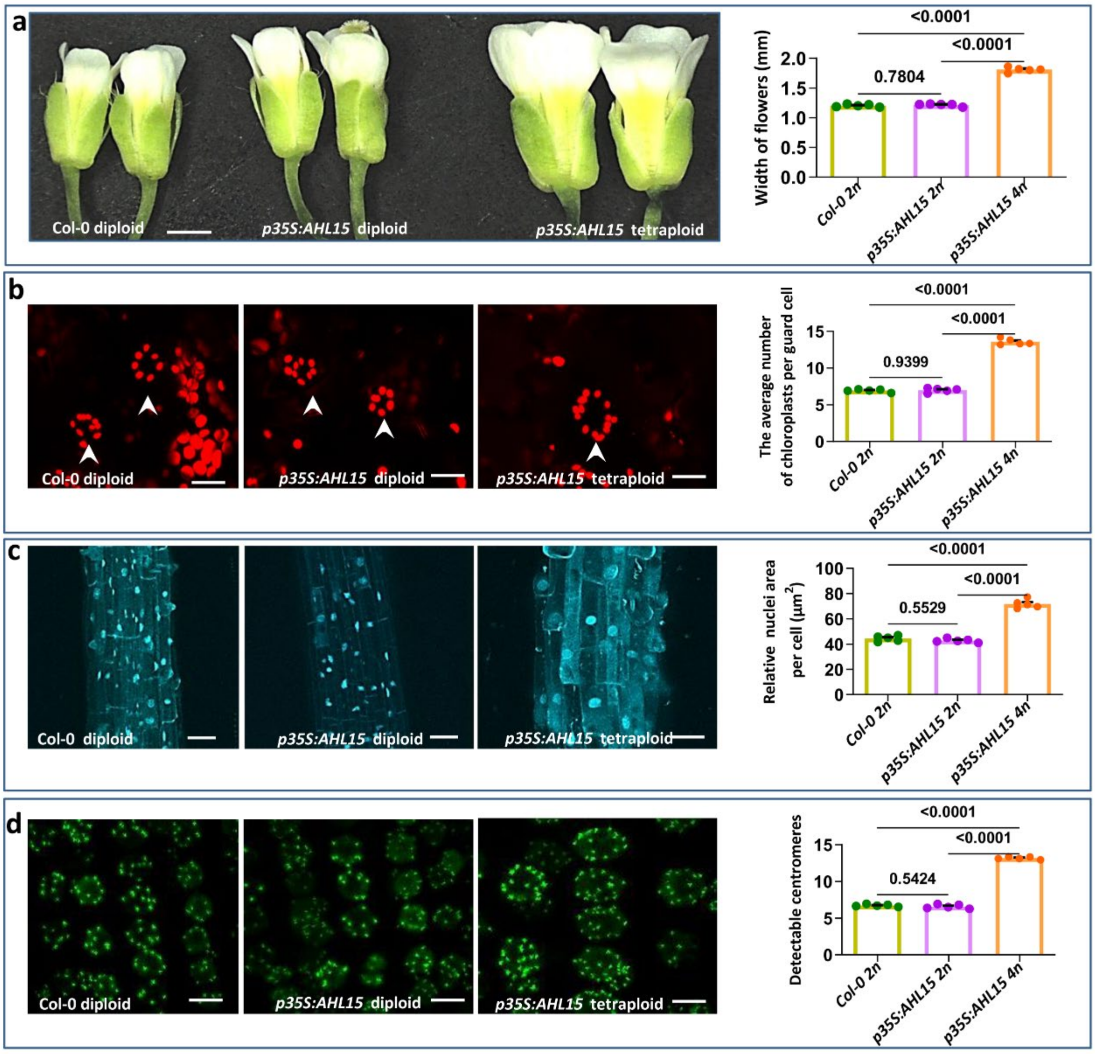

Supplementary Figure 14 Plants regenerated from *p35S:AHL15*-induced somatic embryos are frequently polyploid. a-d Analysis of wild-type *Arabidopsis* (left), and a diploid (middle) and tetraploid (right) plant line each regenerated from an *p35S:AHL15*-induced somatic embryo. **a** Tetraploid *35S:AHL15* plants show increased organ size compared to the diploid control plants, as demonstrated by the size of the flower organs. **b-d** Tetraploid *p35S:AHL15* plants have twice the number of chloroplasts in guard cells (marked by arrow heads (**b**), show root cells with a larger nucleus and cell size (c), and show a duplication in the CENH3-GFP-labelled centromeres per nucleus (**d**) compared to wild-type and diploid *p35S:AHL15 Arabidopsis* plants. Dots in the graphs indicate the values of six biological replicates per plant line with measurement 30 flowers per replicate (in **a**), 30 guard cells per replicate (in **b**) and 30 nuclei per replicate (in **c** and **d**), bar indicates the mean, and error bars the s.e.m. The p-values were determined by a one-way ANOVA with Tukey's honest significant difference post hoc test. The size bar in the images indicates 1mm (**a**), 8 µm (**b**), 22 µm (**c**) and 6 µm (**d**).

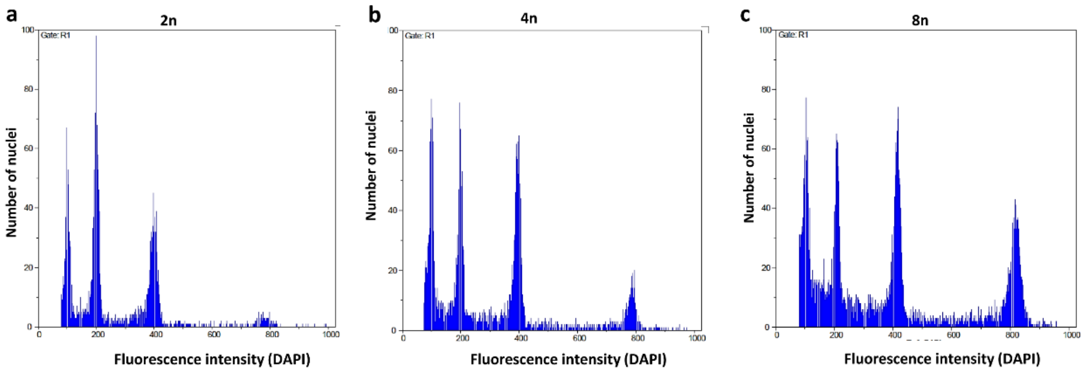

Supplementary Figure 15 Flow cytometry analysis of ploidy on plants derived from *AHL15* overexpression-induced somatic embryos. a-c Flowcytometry histograms produced with a FL2 (480nm) photodetector showing the relative DNA content in DAPI-stained nuclei of mature leaves of a diploid (**a**, 2n), tetraploid (**b**, 4n) and octoploid (**c**, 8n) somatic embryo-derived *p35S:AHL15* plant.

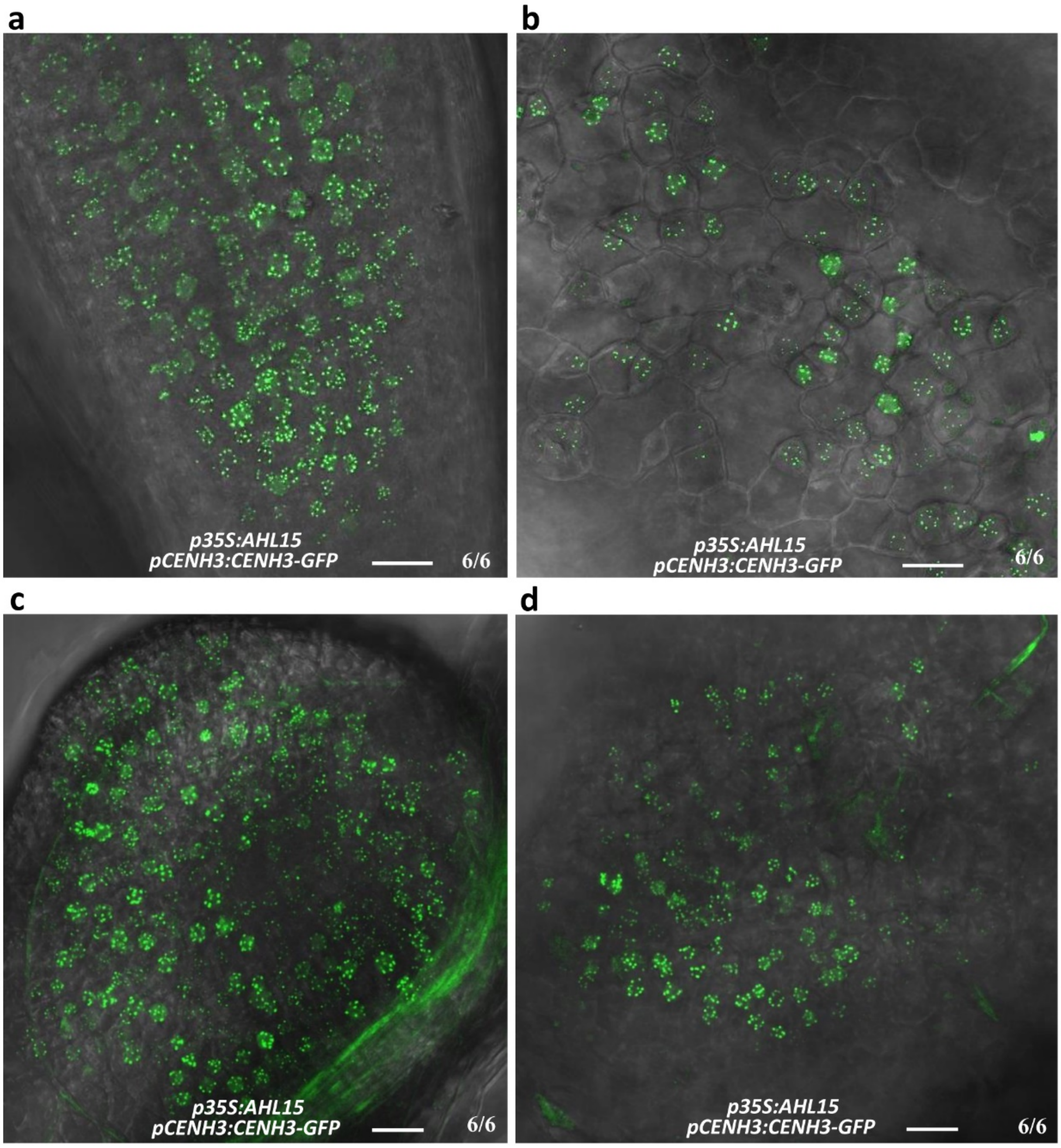

Supplementary Figure 16 Endoploidy does not occur in *p35S:AHL15* leaves and roots. a-d CENH3-GFP–mediated centromere labeling in cells of a root tip (**a**), a young leaf (**b**), or in cells of 2,4-D-induced callus on a root (**c**), or a young leaf (**d**). Size bar indicates 12 µm. Numbers indicate the frequency with which similar results were obtained in 3 independent experiments.

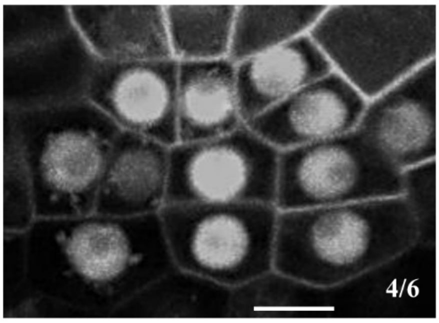

Supplementary Figure 17 Confocal microscopy image of a cotyledon of a *pWOX2:NLS-YFP pAUX1:AUX1-YFP* IZE after 7 days of culture on B5 medium containing 2,4-D. *pWOX2:NLS-YFP* and *pAUX1:AUX1-YFP* reporters were used to mark embryonic nuclei and plasma membranes, respectively. The numbers indicate the frequency with which similar results were obtained in 3 independent experiments. Image shows a merge of the transmitted light and the YFP channel. Size bar indicates 5 μm.

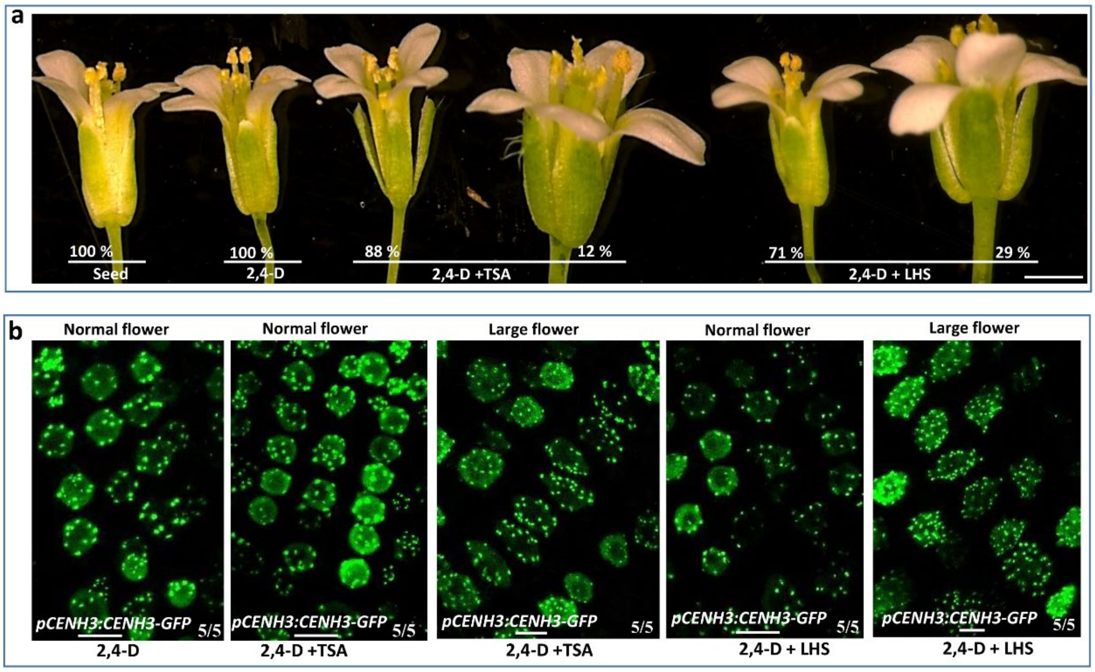

Supplementary Figure 18 Somatic embryo-derived plants obtained after s2,4-D + TSA or 2,4-D + LHS treatment are frequently polyploid. a Percentage of normal and large flower-baring plants derived from Col-0 seeds (left), or derived from somatic embryos induced by 2,4,D alone (2nd left) or by 2,4-D with TSA (middle) or by 2,4-D with long heat stress (LHS). **b** Confocal images showing CENH3-labelled centromeres in root tip cells of the normal or large flower-baring plants shown in (**a**). Numbers indicate the frequency with which similar results were obtained from 2 independent experiments. Size bar indicates 1mm (in **a**), and 5 µm (in **b**).

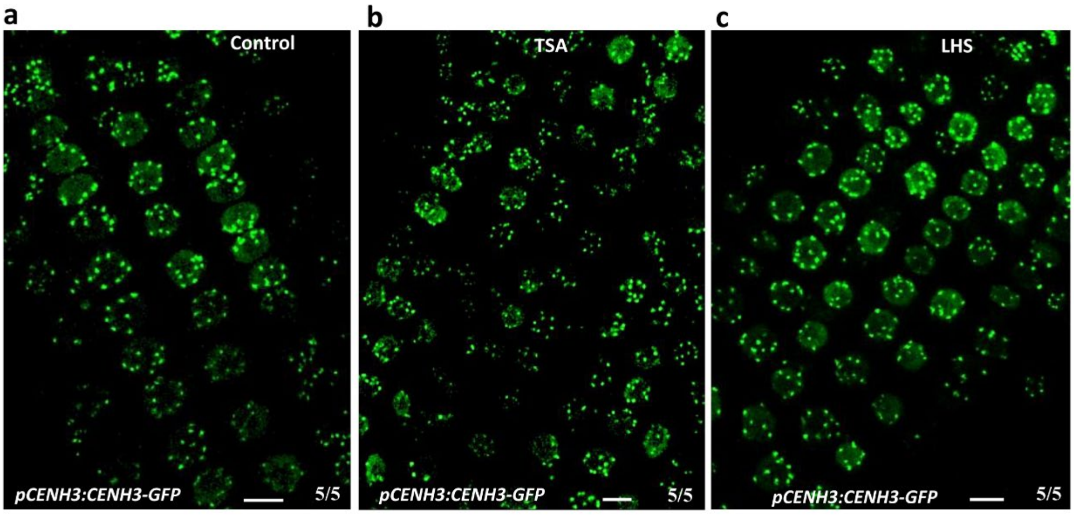

Supplementary Figure 19 Endoploidy does not occur in roots cells treated with TSA or LHS. a-c CENH3-GFP–mediated centromere labeling in untreated root tip cells (control) (**a**), or root tip cells treated 2 days with 1µM TSA (**b**), or for 2 days with LHS (**c**). Numbers indicate the frequency with which similar results were obtained in 2 independent experiments. Size bar indicates 6 µm.

Supplementary Table 1 Primers used for cloning, genotyping and qRT-PCR (F: forward; R: reverse)

Name	Sequence (5' to 3')	Purpose
35S:AHL15-F	CCCGGGATGGCGAATCCTTGGTGGGTAG	*35S:AHL15 construct*
35S:AHL15-R	GGATCCTCAATACGAAGGAGGAGCACG	
35S:AHL29-F	ATAAGAATGCGGCCGCGACGGTGGTTACGATCAATC	*35S:AHL29* construct
35S:AHL29-R	ATAGTTTAGCGGCCGCCTAAAAGGCTGGTCTTGGTG	
35S:AHL20 –F	ATAAGAATGCGGCCGCGCAAACCCTTGGTGGACGAAC	*35S:AHL20* construct
35S:AHL20-R	ATAGTTTAGCGGCCGCTCAGTAAGGTGGTCTTGCGT	
35S:AHL19-F	GGGGACAAGTTTGTACAAAAAAGCAGGCTCGATGGCGAATCCATGGTGGAC	*35S:AHL19 construct*
35S:AHL19-R	GGGGACCACTTTGTACAAGAAAGCTGGGTAAACAAGTAGCAACTGACTGG	
pAHL15-GUS-F	GGGGACAAGTTTGTACAAAAAAGCAGGCTCGACACTCCTCTGTGCCACATT	*pAHL15:AHL15-GUS* construct
pAHL15-GUS-R	GGGGACCACTTTGTACAAGAAAGCTGGGTAATACGAAGGAGGAGCACGAG	*pAHL15:AHL15-tagRFP*
I miR-s AHL20	GATTAGACTACCTCAAATTGCTATCTCTCTTTTGTATTCC	
II miR-a AHL20	GATAGCAATTTGAGGTAGTCTAATCAAAGAGAATCAATGA	
III miR*s AHL20	GATAACAATTTGAGGAAGTCTATTCACAGGTCGTGATATG	*35S:amiRAHL20* construct
IV miR*a AHL20	GAATAGACTTCCTCAAATTGTTATCTACATATATATTCCT	
amiRNA AHL20-F	GGGGACAAGTTTGTACAAAAAAGCAGGCTCGCGACGGTATCGATAAGCTTG	
amiRNA AHL20-R	GGGGACCACTTTGTACAAGAAAGCTGGGTACCCATGGCGATGCCTTAAAT	
SALK_040729-F	GTCGGAGAGCCATCAACACCA	ahl15 genotyping
SALK_040729-R	CGACGACCCGTAGACCCGGATC	
SALK_070123-F	GGCGAATCCATGGTGGACAGG	ahl19 genotyping
SALK_070123-R	GGCCGCTCATCTGTCCTCCTC	
qAHL15-F	AAGAGCAGCCGCTTCAACTA	qRT-PCR *AHL15*
qAHL15-R	TGTTGAGCCATTTGATGACC	
qAHL20-F	CAAGGCAGGTTTGAAATCTTATCT	qRT-PCR *AHL120*
qAHL20-R	TAGCGTTAGAGAAAGTAGCAGCAA	
qAHL19-F	CTCTAACGCGACTTACGAGAGATT	qRT-PCR *AHL19*
qAHL19-R	ATATTATACACCGGAAGTCCTTGGT	
qβ-TUBULIN-6-F	TGGGAACTCTGCTCATATCT	qRT-PCR *TUBULIN-6*
qβ-TUBULIN-6-R	GAAAGGAATGAG GTTCACTG	
qSAND-F	AACTCTATGCAGCATTTGATCCACT	qRT-PCR *SAND*
qSAND-R	TGATTGCATATCTTTATCGCCATC	
