# Supplementary material for: An Arabidopsis AT-hook motif nuclear protein mediates somatic embryogenesis and coinciding genome duplication
Source: Nat Commun. 2021 May 4;12:2508. doi: 10.1038/s41467-021-22815-8 (PMC8096963; doi:10.1038/s41467-021-22815-8)
Supplement: Supplementary file 3 — Reporting Summary [file 41467_2021_22815_MOESM3_ESM.pdf]

natureresearch

Corresponding author(s): Omid Karami and Remko Offringa

Last updated by author(s): Mar 29, 2021

Reporting Summary

Nature Research wishes to improve the reproducibility of the work that we publish. This form provides structure for consistency and transparency in reporting. For further information on Nature Research policies, see Authors & Referees and the Editorial Policy Checklist .

Statistics

For all statistical analyses, confirm that the following items are present in the figure legend, table legend, main text, or Methods section.

n/a	Confirmed	
☐	☒	The exact sample size (*n*) for each experimental group/condition, given as a discrete number and unit of measurement
☐	☒	A statement on whether measurements were taken from distinct samples or whether the same sample was measured repeatedly
☐	☒	The statistical test(s) used AND whether they are one- or two-sided<br>*Only common tests should be described solely by name; describe more complex techniques in the Methods section.*
☒	☐	A description of all covariates tested
☒	☐	A description of any assumptions or corrections, such as tests of normality and adjustment for multiple comparisons
☐	☒	A full description of the statistical parameters including central tendency (e.g. means) or other basic estimates (e.g. regression coefficient) AND variation (e.g. standard deviation) or associated estimates of uncertainty (e.g. confidence intervals)
☐	☒	For null hypothesis testing, the test statistic (e.g. *F*, *t*, *r*) with confidence intervals, effect sizes, degrees of freedom and *P* value noted<br>*Give P values as exact values whenever suitable.*
☒	☐	For Bayesian analysis, information on the choice of priors and Markov chain Monte Carlo settings
☒	☐	For hierarchical and complex designs, identification of the appropriate level for tests and full reporting of outcomes
☒	☐	Estimates of effect sizes (e.g. Cohen's *d*, Pearson's *r*), indicating how they were calculated

Our web collection on statistics for biologists contains articles on many of the points above.

Software and code

Policy information about availability of computer code

Data collection	Image J
Data analysis	Image J

For manuscripts utilizing custom algorithms or software that are central to the research but not yet described in published literature, software must be made available to editors/reviewers. We strongly encourage code deposition in a community repository (e.g. GitHub). See the Nature Research guidelines for submitting code & software for further information.

Data

Policy information about availability of data

All manuscripts must include a data availability statement. This statement should provide the following information, where applicable:

- Accession codes, unique identifiers, or web links for publicly available datasets
- A list of figures that have associated raw data
- A description of any restrictions on data availability

All data generated or analysed during this study are included in this published article (and its supplementary information files).

Field-specific reporting

Please select the one below that is the best fit for your research. If you are not sure, read the appropriate sections before making your selection.

☒ Life sciences ☐ Behavioural & social sciences ☐ Ecological, evolutionary & environmental sciences

For a reference copy of the document with all sections, see nature.com/documents/nr-reporting-summary-flat.pdf

Life sciences study design

All studies must disclose on these points even when the disclosure is negative.

Sample size	No statistical analysis was used to determine sample size. Sample size was chosen based on what is common in the field
Data exclusions	No data have been excluded from our analysis
Replication	All described experiments were repeated at least once, and appeared reproducible.
Randomization	For plant phenotyping and morphometry, progeny of a genetically homozygous plant parent were randomly selected.
Blinding	Blinding was not relevant in our study, since the phenotypic differences between the different lines were sufficiently large.

Reporting for specific materials, systems and methods

We require information from authors about some types of materials, experimental systems and methods used in many studies. Here, indicate whether each material, system or method listed is relevant to your study. If you are not sure if a list item applies to your research, read the appropriate section before selecting a response.

Materials & experimental systems

n/a	Involved in the study	
☐	☒	Antibodies
☒	☐	Eukaryotic cell lines
☒	☐	Palaeontology
☒	☐	Animals and other organisms
☒	☐	Human research participants
☒	☐	Clinical data

Methods

n/a	Involved in the study	
☐	☒	ChIP-seq
☒	☐	Flow cytometry
☒	☐	MRI-based neuroimaging

Antibodies

Antibodies used	1. Anti-dimethyl-Histone H3 (Lys9) Antibody, Sigma Aldrich, Catalog number 07212, Lot # 3243503<br>2. Anti-Rabbit IgG (H+L), CF™488A antibody produced in F(ab')2 fragment of goat, Sigma Aldrich, Catalog number SAB4600234-125UL, Lot # 19C0507
Validation	1. https://www.sigmaaldrich.com/catalog/product/mm/07212?lang=en&region=NL<br>2. https://www.sigmaaldrich.com/catalog/product/sigma/sab4600234?lang=en&region=NL

ChIP-seq

Data deposition

☒ Confirm that both raw and final processed data have been deposited in a public database such as GEO.

☒ Confirm that you have deposited or provided access to graph files (e.g. BED files) for the called peaks.

Data access links<br>*May remain private before publication.*	https://www.ncbi.nlm.nih.gov/geo/query/acc.cgi?acc=GSE52400
Files in database submission	GSM1264686 pBBM::BBM-YFP, GSM1264687 pBBM::NLS-GFP, GSM1264688 35S::BBM-GFP, GSM1264689 35S::BBM
Genome browser session<br>(e.g. UCSC)	n/a, as the ChIP details have previously been published in Horstman et al. 2015, Development 142, 454-464

Methodology

Replicates	*Describe the experimental replicates, specifying number, type and replicate agreement.*
Sequencing depth	n/a
Antibodies	n/a

Peak calling parameters	n/a
Data quality	n/a
Software	n/a, as the ChIP details have previously been published in Horstman et al. 2015, Development 142, 454-464
